# Charge Transfer Reactions between Water Isotopologues and Kr^+^ ions

**DOI:** 10.1021/acsphyschemau.1c00042

**Published:** 2022-01-31

**Authors:** Andriana Tsikritea, Jake A. Diprose, Jérôme Loreau, Brianna R. Heazlewood

**Affiliations:** † Department of Chemistry, University of Oxford, Physical and Theoretical Chemistry, South Parks Road, Oxford, OX1 3QZ, United Kingdom; ‡ Department of Physics, University of Liverpool, Liverpool, L69 7ZE, United Kingdom; § KU Leuven, Department of Chemistry, Celestijnenlaan 200F, Leuven, B-3001, Belgium

**Keywords:** Coulomb crystals, reaction kinetics, *ab initio* calculations, capture theory models, astrochemistry

## Abstract

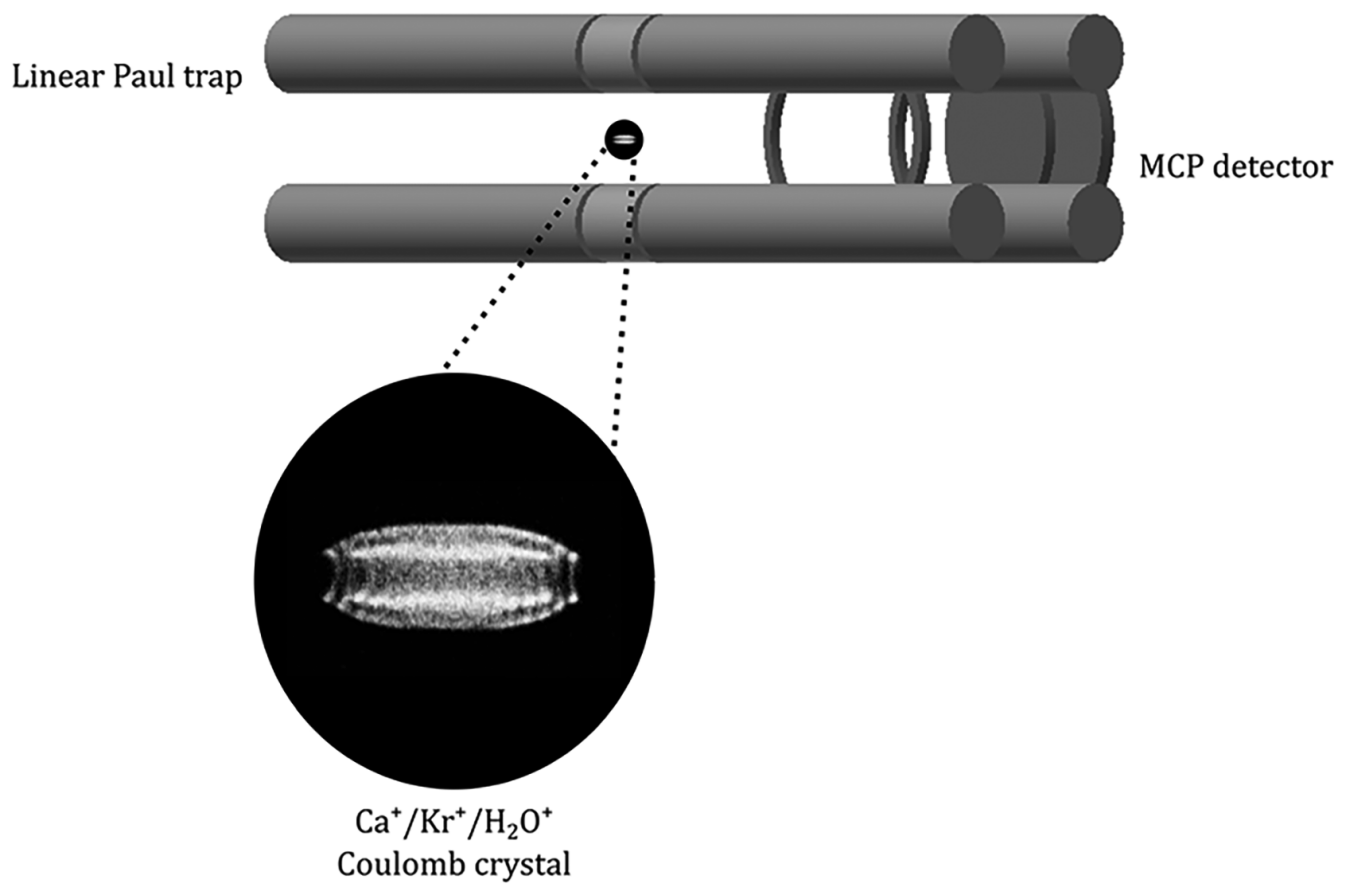

Astrochemical models often adopt capture theories to predict the behavior of experimentally unmeasured ion–molecule reactions. Here, reaction rate coefficients are reported for the charge transfer reactions of H_2_O and D_2_O molecules with cold, trapped Kr^+^ ions. Classical capture theory predictions are found to be in excellent agreement with the experimental findings. A crossing point identified between the reactant and product potential energy surfaces, constructed from high-level *ab initio* calculations, further supports a capture-driven mechanism of charge transfer. However, ion–molecule reactions do not always agree with predictions from capture theory models. The appropriateness of using capture theory-based models in the absence of detailed experimental or theoretical studies is discussed, alongside an analysis of why capture theory is appropriate for describing the likelihood of charge transfer between Kr^+^ and the two water isotopologues.

## Introduction

More than 200 molecular species have now been unambiguously identified in the interstellar medium (ISM),^1^ and hundreds of deuterated analogues of these molecules have also been observed.^2^ Ion–molecule reactions are expected to play an important role in the gas-phase chemistry occurring in the ISM, as these reactions often exhibit a negative trend with temperature, with enhanced rates at low temperatures.^3,4^ Spectroscopic observations have been critical in identifying species that are present in the ISM. However, in order to understand the chemistry of the ISM and to establish how the detected molecular species were formed, more details are required. To unravel the complex chemistry of the ISM, a collaborative effort is needed—combining laboratory studies and the development of detailed models with spectroscopic observations and astrochemical databases. There is a well-acknowledged lack of experimental data recorded under conditions relevant to the ISM, especially when considering deuterated analogues. As such, capture theories are often employed in astrochemical models to provide an estimate of the rate coefficients exhibited by experimentally unmeasured ion–molecule reactions.^5^

Capture theories have been found to successfully predict a number of experimentally measured rate coefficients over a temperature range that spans from >300 K down to 10 K.^6^ However, there are also examples of reaction systems for which capture theories cannot account for the likelihood of reaction. Such behavior was observed in the charge transfer of ammonia isotopologues with rare-gas ions (Xe^+^, Kr^+^, and Ar^+^)—with the experimental rate coefficients consistently lower than those predicted by capture theory.^7,8^ The charge transfer processes also exhibited an unexpected inverse kinetic isotope effect (KIE): for all three rare-gas ions studied, ND_3_ reacted faster than NH_3_. Ammonia is known to be present in the ISM, with both ND_3_ and NH_3_ spectroscopically identified.^9^ Water is another small polar molecule found in the ISM, with both H_2_O and D_2_O identified.^10^ To establish whether kinetic isotope effects might be generally observed in charge transfer reactions involving rare-gas ions and small polar molecules, and to test the accuracy of capture theory in additional astrochemically relevant reaction systems, we considered the reactivity of water isotopologues.

In this work, we examine charge transfer reactions between thermal water (H_2_O or D_2_O) molecules and cold, trapped Kr^+^ ions. In both systems, the experimental reaction rate coefficients are found to be in good agreement with predictions from capture theory models. No kinetic isotope effects are observed; H_2_O and D_2_O exhibit very similar rate coefficients. Charge transfer therefore appears to be a capture-limited process, displaying a dependence on the strength of the long-range attractive forces between the neutral polar molecule and the ionic reactant. The success of capture theory models in describing the behavior of ion–molecule interactions is discussed—both in the context of the findings detailed in this work, and in more general terms.

## Methods

### A Experimental Section

**Figure 1:**
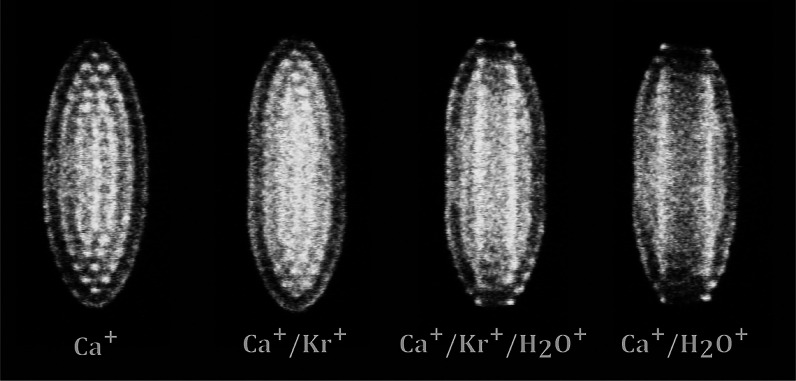
Coulomb crystal images at different times during a charge transfer reaction between Kr^+^ ions and H_2_O neutrals. Water molecules introduced to the ion trap volume initiate the reaction. The formation and growth of a dark core qualitatively shows the reaction progress.

Charge transfer reactions are studied within Ca^+^ Coulomb crystals, following the same approach adopted in previous work.^7,8^ Briefly, Ca atoms, produced by a resistively heated oven, are nonresonantly ionized at the center of a linear Paul ion trap using the 355 nm output of a frequency-tripled Nd:YAG laser. The resulting Ca^+^ ions are dynamically confined by a combination of radiofrequency and static voltages applied to the four cylindrical trap rods. A closed-cycle laser cooling scheme is implemented, with a 397 nm diode driving the 4s^2^S_1/2_ → 4p^2^P_1/2_ transition and a 866 nm diode returning population (lost to the ^2^D_3/2_ state) to the main cooling transition by exciting the 3d^2^D_3/2_ → 4p^2^P_1/2_ transition. Once sufficient kinetic energy has been removed,^11^ laser-cooled Ca^+^ ions can form Coulomb crystals—adopting a regular 3-dimensional lattice-like structure. Fluorescence emitted as part of the laser cooling cycle is captured by a charge-coupled device (CCD) camera and 10× microscope objective lens. Figure 1 depicts Ca^+^ Coulomb crystal images captured at different times during a charge transfer reaction. Sympathetically cooled species (i.e., co-trapped ions that are translationally cold due to elastic collisions with the laser-cooled Ca^+^ ions) do not fluoresce. However, the presence of co-trapped ions induces changes in the locations of the fluorescing Ca^+^ ions within the Coulomb crystal, and these changes can be directly observed by the CCD camera in real time. Time-of-flight mass spectra (ToF-MS) are recorded by ejecting the ions toward a flight tube and onto a microchannel plate (MCP) detector.^12^

Kr atoms are introduced to the reaction chamber through a high-precision leak valve interfaced with a three-way pulsed valve system, admitting a constant and reproducible volume of gas.^13^ A (2 + 1) REMPI scheme at 212.6 nm, using the tripled output of a Nd:YAG-pumped dye laser, forms Kr^+^ ions in the trap center. A time delay of ∼15 s is introduced to allow for sympathetic cooling of the Kr^+^ ions into the crystal, and to ensure that all Kr^+^ ions are in the ground electronic state.^8,11^ Any Kr^+^ ions produced in the higher energy ^2^P_1/2_ spin state have a lifetime of only 340 ms, decaying to the ground ^2^P_3/2_ state via a magnetic-dipole-allowed transition with an Einstein coefficient of *A*_*m*_ = 2.8 s^–1^.^14^

H_2_O or D_2_O vapor at 293 K is admitted to the reaction chamber through a second high-precision leak valve, initiating the charge transfer reactions. The product ions form a dark core, the growth of which indicates the reaction progress (see Figure 1). Experimental images are compared with molecular dynamics simulations, yielding quantitative information on the crystals’ composition as a function of the reaction time. To complement the imaging analysis, crystals are ejected from the ion trap at selected reaction times and ToF-MS traces are recorded—thereby confirming the mass-to-charge ratios and relative abundances of all ions in the crystal at the point of ejection. No competing reaction pathways are observed. Charge transfer is found to be a one-to-one process^15^ that follows a pseudo-first order kinetic model.^7,8^ Bimolecular rate coefficients are calculated assuming a constant water partial pressure, with the partial pressure established by calibrating the high-precision leak valve to an ion gauge and a residual gas analyzer (see Supporting Information for further details).

### B Potential Energy Surfaces

Potential energy surfaces (PESs) for the H_2_O–Kr^+^ system have been constructed by means of the multiconfigurational self-consistent field method and the multireference configuration interaction (MRCI) method including the Davidson correction, as implemented in the MOLPRO2018.1 quantum chemistry package.^16−18^

H_2_O in its ground electronic state (X^1^A_1_) has an HOH angle of θ_HOH_ = 104.5° and a bond length *R*_OH_ = 0.9576 Å. For the cation H_2_O^+^ in its ground state (X̃ ^2^B_1_), θ_HOH_ = 110.46° with bond length *R*_OH_ = 0.9988 Å. Due to the similarity in geometries, the O–H bond length and the HOH angle are kept fixed at the equilibrium value of H_2_O in the present calculations. This leads to three-dimensional PESs, as discussed below.

The initial state, Kr^+^(^2^P) + H_2_O(^1^A_1_), gives rise to two ^2^A′ states and a ^2^A″ state in the C_s_ group, while the exit channel, Kr(^1^S) + H_2_O^+^(X̃ ^2^B_1_), leads to a ^2^A″ state in C_s_, for a total of two ^2^A′ states and two ^2^A″ states (or four states in the C_1_ group). We note that the first excited state, H_2_O^+^(Ã^2^A_1_), has an ionization potential about 0.15 eV higher than Kr, and could therefore potentially be reached. However, the geometry of H_2_O in this state is nearly linear (with bond length *R*_OH_ = 0.98 Å)—a very different geometry to what is seen in ground state H_2_O^+^ and ground state H_2_O. As such, the excited Ã^2^A_1_ state is neglected in the present calculations.

The basis set used is aug-cc-pVTZ for all atoms. For the Kr atom the 10 innermost electrons are described by an effective core potential (ECP10MDF); the 3s^2^3p^6^3d^10^ orbitals are kept frozen. For the O atom the 1s^2^ orbitals are frozen. The active space consists of the remaining orbitals, that is, 4s^2^4p^6^ for Kr, 2s^2^2p^4^ for O, and 1s for each H atom. In the C_s_ point group, this leads to 7a′ and 3a″ core orbitals, with 7a′ and 3a″ additional orbitals in the active space. The spin–orbit splitting in Kr^+^ (5370 cm^–1^) is taken into account by diagonalizing the Breit–Pauli operator on the basis of MRCI wave functions.

## Results

### Reaction Rate Coefficients

The experimental rate coefficients measured for the Kr^+^ + H_2_O and Kr^+^ + D_2_O reaction systems are *k*_H_ = 1.8(7) × 10^–9^ cm^3^ s^–1^ and *k*_D_ = 2(1) × 10^–9^ cm^3^ s^–1^, respectively. The reactions are assigned an effective temperature of 244 K for the Kr^+^ + H_2_O system and 240 K for the Kr^+^ + D_2_O system. This temperature represents a weighted average of the thermal water reactants (293 K in our laboratory) and the sympathetically cooled Kr^+^ ions (at ≈15 K). The latter is estimated from molecular dynamics simulations and accounts for both the secular motion and the micromotion of the trapped ions.

The average dipole orientation (ADO) capture theory model is employed for comparison with the experimentally measured rate coefficients. ADO theory describes the interaction between ions and polar molecules in a capture framework, where any collision that leads to complex formation proceeds to products with unit probability. The model assumes a classical distribution of rotational states for the neutral reactant.^19,20^ As our reactions take place at temperatures around 240 K, ADO is expected to approximately predict the rate of capture. Further details on the ADO calculations are provided in previous work;^7,8^ the constants and input parameters used in this work are set out in Table 2.

**Figure 2:**
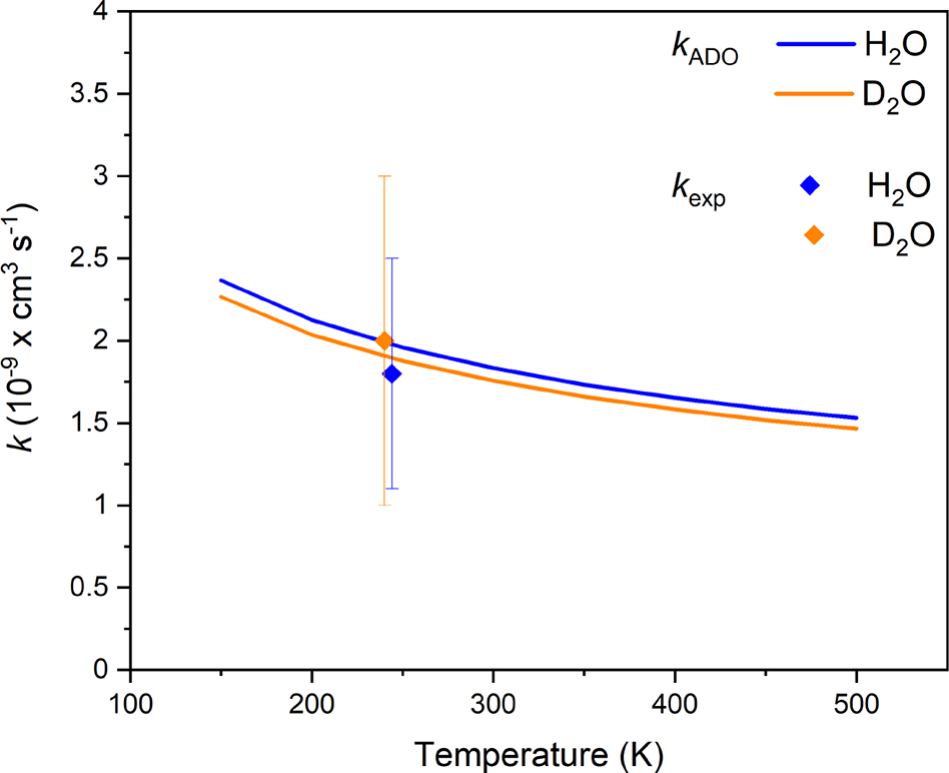
Charge transfer rate coefficients for the Kr^+^ + H_2_O (blue) and Kr^+^ + D_2_O (orange) reaction systems, as established from ADO capture theory models and from experimental measurements. Error bars indicate the uncertainty in the experimental rate coefficients (see Supporting Information for further details).

Experimental rate coefficients are set out in Table 1 and Figure 2, alongside the predicted values from the ADO calculations. Very similar rate coefficients are observed for the two isotopologues; Kr^+^ ions react with H_2_O and D_2_O at a comparable rate. The experimental rate coefficients are in excellent agreement with the classical ADO theory calculations. As the reactions are studied under conditions for which classical descriptions hold, this agreement indicates that electron transfer is a capture-limited process. Other capture theory models are also consistent with the experimental rate coefficients. Details of these calculations are given in the Supporting Information. The relative magnitudes of the experimental rate coefficients given in Table 1 suggest that no KIE is present, with *k*_H_/*k*_D_ = 0.9(6).

**Table 1:** Experimental and ADO Rate Coefficients for the Charge Transfer Reactions between Kr^+^ and Two Water Isotopologues^a^

reaction system	*k*_exp_ (cm^3^ s^–1^ × 10^–9^)	*k*_H_/*k*_D_	*k*_ADO_ (cm^3^ s^–1^ × 10^–9^)	*T* (K)
Kr^+^ + H_2_O	1.8(7)		1.98	244
Kr^+^ + D_2_O	2(1)	0.9(6)	1.90	240
Kr^+^ + NH_3_	0.51(5)^b^		1.82	246
Kr^+^ + ND_3_	1.0(3)^b^	0.5(2)	1.69	239

a Findings reported in previous work, for the Kr^+^ + ammonia reaction systems, are also given. Uncertainties are stated in parentheses (see Supporting Information for details on how the uncertainties are established), with the effective reaction temperatures specified.

b Tsikritea et al.^8^

**Table 2:** Polarizabilities,^30−32^ Dipole Moments,^30,32,33^ and *c* Parameters^20^ Used to Calculate the ADO Rate Coefficients

isotopologue	α (cm^3^)	μ_D_ (D)	*c*
H_2_O	1.45 × 10^–24^	1.848	0.254
D_2_O	1.44 × 10^–24^	1.851	0.254
NH_3_	2.16 × 10^–24^	1.47	0.227
ND_3_	1.88 × 10^–24^	1.50	0.232

**Figure 3:**
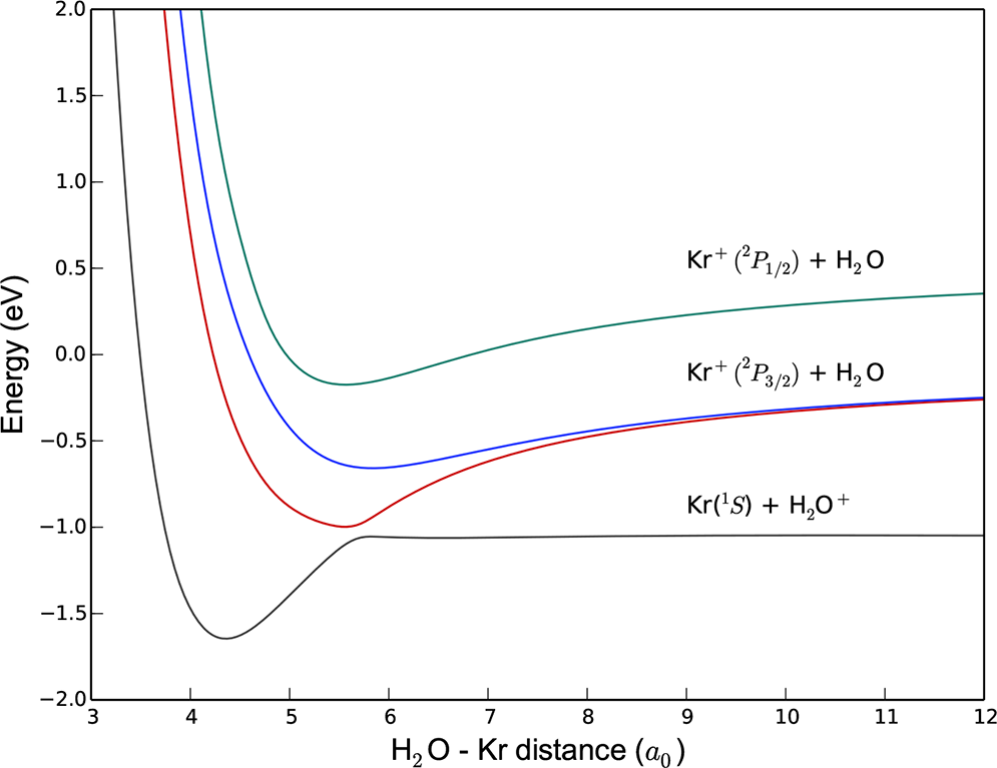
One-dimensional cut of the PESs of the [H_2_OKr]^+^ complex participating in the charge transfer process, as a function of the distance *R* between the Kr atom and the center of mass of H_2_O. The angles are θ = ϕ = 0°, corresponding to the Kr atom approaching along the negative side of the dipole of H_2_O.

**Figure 4:**
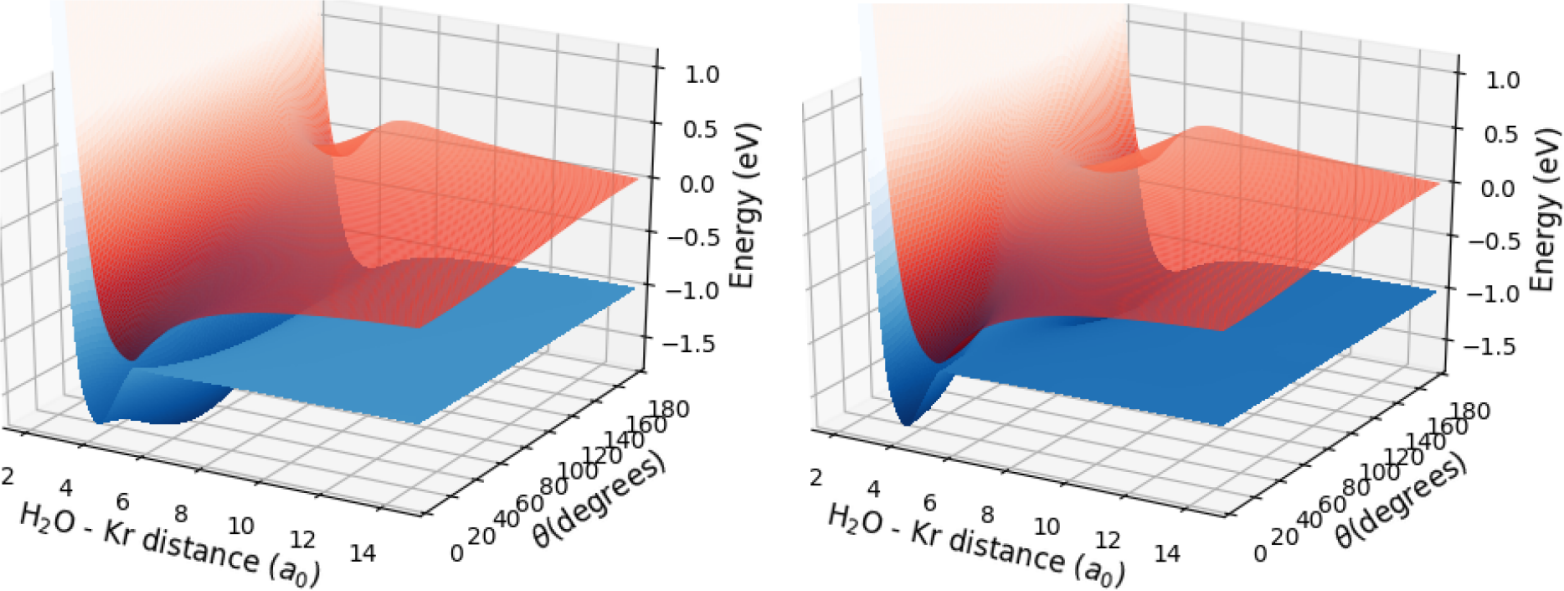
Two-dimensional PESs *V*(*R*, θ) of the lowest two electronic states of the [H_2_OKr]^+^ complex participating in the charge transfer process. Left panel, ϕ = 0°; right panel, ϕ = 90°.

These experimental results can be understood on the basis of three-dimensional PESs *V*(*R*, θ, ϕ), where *R* is the length of the vector **R** describing the position of the Kr atom (ion) with respect to the center of mass of H_2_O^(+)^, θ is the angle between the vector **R** and the C_2_ axis of H_2_O, and ϕ is the angle of rotation of this vector around the C_2_ axis. The PESs have been constructed for several values of the angle ϕ by computing the energy for 26 values of the internuclear distance *R* between 3 *a*_0_ and 15 *a*_0_, and 11 values of θ between 0° and 180°. The most favorable approach for H_2_O + Kr^+^ collisions is when θ = 0°, where the ion approaches along the negative end of the dipole vector. The potential energy curves for this orientation are shown in Figure 3. We observe the presence of a strong avoided crossing between the entrance and exit channels at a distance of 5.7*a*_0_, leading to an efficient charge transfer process. Collisions between the reactants at other orientations are less favorable. Two-dimensional PESs for the two lowest electronic states shown in Figure 3 are illustrated for ϕ = 0° and ϕ = 90° in Figure 4.

### Other Reaction Pathways

Charge transfer is the only energetically accessible reaction pathway under the experimental conditions. The formation of ground-state H_2_O^+^ ions, Kr^+^(^2^P_3/2_) + H_2_O → Kr + H_2_O^+^(X̃ ^2^B_1_), has a reported exothermicity of Δ*H* = −1.38 eV.^15^ All other reaction pathways, such as the hydrogen abstraction channel Kr^+^(^2^P_3/2_) + H_2_O → KrH^+^ + OH (Δ*H* = 0.4 eV), are endothermic.^15^ ToF-MS data confirm the absence of any competitive reaction channels, with only charge transfer product ions observed. While we are not aware of any previous studies on the Kr^+^ + D_2_O reaction system, the absence of any KrD^+^ ions in the ToF-MS traces suggests that this channel is also energetically closed for deuterated water. Most background gases in the reaction chamber are removed by a heat exchange device.^7,8^ Any remaining species are identified with a residual gas analyzer (RGA). Under the experimental conditions, reactions between Kr^+^ ions and background species present in the reaction chamber are typically absent or negligible. If any such reactions do occur, they are monitored by ToF-MS, quantified using molecular dynamics simulations, and included in the kinetic model used to extract reaction rate coefficients. A detailed discussion on the treatment of background reactions can be found in previous work.^7,8^ Specific to this study, the possibility of hydrogen exchange with D_2_O in the liquid reservoir (prior to admission of the reactant to the ion trap volume) is carefully monitored throughout the measurements using the RGA. The reservoir is refilled frequently, with the RGA confirming that there is minimal hydrogen exchange.

It should also be noted that Ca^+^ ions within the Coulomb crystal—the laser-cooled species that sympathetically cool co-trapped ions—can themselves react with water to produce CaOH^+^ (or CaOD^+^) ions. The reaction involves Ca^+^ ions in the metastable 3d^2^D_3/2_ state, populated as part of the laser cooling cycle.^21^ To ensure that any side reactions are accurately taken into account, extensive background reaction studies between monocomponent Ca^+^ Coulomb crystals and water neutrals (both H_2_O and D_2_O) have been conducted. Under the experimental conditions adopted in this work, the rate at which Ca^+^ ions react with water is negligible on the time scale of the charge transfer reactions. On the few occasions when CaOH^+^ or CaOD^+^ ions are detected in the ToF traces, they are included in the analysis and explicitly taken into account.

## Discussion

### Reaction Mechanism

The Kr^+^ + H_2_O reaction has been previously studied at 296 K using the flowing afterglow technique, with a charge transfer rate coefficient of *k* = 1.2(2) × 10^–9^ cm^3^ s^–1^ reported.^22^ It is unclear, however, if the Kr^+^ ions in this earlier study were exclusively in the ground ^2^P_3/2_ electronic state (as in this work), or whether a mixture of electronic states (^2^P_3/2_ and ^2^P_1/2_) were populated. Additionally, the flowing afterglow experiments were carried out under different conditions to those adopted in this work—at pressures several orders of magnitude higher, at a higher collision energy, and with a different distribution of internal energy in the reactants. As such, it is not possible to directly compare the rate coefficients reported from the earlier flowing afterglow study with the findings of this work.

Another study, undertaken at hyperthermal collision energies (spanning 0.1–10 eV) in an octopole guided ion-beam apparatus, did consider the reactivity of Kr^+^(^2^P_3/2_) and Kr^+^(^2^P_1/2_) separately.^15^ While the collision energies in the hyperthermal study are clearly very different to those employed in this work, the mechanistic insights that were reported are interesting. For the reaction of Kr^+^(^2^P_3/2_) with H_2_O, only ground state water ion products, H_2_O^+^(X̃^2^B_1_), were detected. In contrast, “intense” luminescence was observed following the reaction of spin–orbit excited Kr^+^(^2^P_1/2_) with H_2_O; excited state H_2_O^+^(Ã^2^A_1_) products were formed, with the luminescence arising from the Ã^2^A_1_–X̃^2^B_1_ transition in H_2_O^+^. These observations were rationalized by the significant spin–orbit splitting between the ^2^P_3/2_ and ^2^P_1/2_ states of Kr^+^ and the different Franck–Condon overlap between the reactant and product wave functions. For example, the strongest Franck–Condon factors were identified for the (near-resonant) Kr^+^(^2^P_1/2_) + H_2_O reactants yielding vibrationally excited H_2_O^+^(Ã^2^A_1_) + Kr products.^15^ Spin–orbit selectivity—where, depending on the spin–orbit state of the rare gas ion, H_2_O^+^ product ions were formed exclusively in the ground or excited electronic state following charge transfer—was also observed in an earlier study involving Ar^+^ ions.^23^ At the lowest collision energies examined (0.1–0.2 eV), charge transfer cross section measurements for the reaction of Kr^+^ with H_2_O were found to be in good agreement with both the ADO and parametrized Su capture theory models.

Earlier photoionization spectroscopic studies identified that an electron is removed from the *p*-type non-bonding *b*_1_ valence orbital centered on the O atom when ground state H_2_O^+^(X̃^2^B_1_) ions are formed.^24^ The *p*-type orbital is oriented out of the molecular plane, and not along the C_2_ axis. Here, *ab initio* calculations identify the most favorable angle of approach for Kr^+^ ions to extract an electron from water as occurring when θ = ϕ = 0° (i.e., along the C_2_ axis)—where the cation approaches along the dipole axis of the water molecule. To confirm the validity of the reaction mechanism identified from the *ab initio* calculations, additional experimental measurements could be conducted. For example, controlling the orientation of the H_2_O reactants through the application of an external electric field (as recently reported for ammonia molecules)^25,26^ would enable the stereodynamics of the reaction to be established.

### Comparison with Other Ion–Molecule Reaction Systems

There have been a number of previous studies into ion–molecule reaction systems under comparable experimental conditions. The astrochemically interesting reaction between sympathetically cooled CCl^+^ ions and thermal CH_3_CN neutrals was recently studied within Ca^+^ Coulomb crystals, at an effective reaction temperature of 160 K. The experimental rate coefficient was found to be in agreement with predictions from ADO capture theory.^27^ For the reaction of non-polar C_2_H_2_ with CCl^+^ ions, the experimental results were successfully described by the Langevin capture model.^28^ The relative reactivity of *ortho*- and *para*-water with sympathetically cooled N_2_H^+^ ions has also been studied using Coulomb crystals. *Para*-water was found to react faster than *ortho*-water, owing to differences in the dipole moment—as explained within the framework of state-selected, rotationally adiabatic quantum capture theory.^29^ Capture theories have also successfully described the behavior of ion–molecule reactions studied using methods such as CRESU (cinétique de réaction en ecoulement supersonique uniforme, or reaction kinetics in uniform supersonic flow). For example, CRESU measurements performed over a range of low temperatures found the N^+^ + H_2_O and N^+^ + NH_3_ reaction systems to be well-described by capture theory models.^4^

The experimental rate coefficients measured in this work for the electron transfer between H_2_O or D_2_O and Kr^+^ are in excellent agreement with ADO capture theory predictions. As highlighted in the preceding paragraph, capture theories have successfully predicted rate coefficients for a range of ion–molecule reaction systems studied under conditions comparable to those adopted in this work. However, this has not always been the case. Recent experimental work on the reactions of ammonia (NH_3_ and ND_3_) with rare-gas ions (including Kr^+^) found charge transfer to be far slower than capture theory models predicted.^7,8^ An inverse KIE was also observed in the charge transfer between ammonia and rare-gas ions—whereas no KIE is seen in the charge transfer reactions studied in this work.

ADO capture theory rate coefficients can be calculated (in SI units) using the following equation^7^
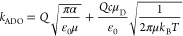
1where *Q* is the charge on the ion, α is the polarizability of the neutral reactant, ε_0_ is the permittivity of free space, μ is the reduced mass of the ion–neutral reaction system, μ_D_ is the dipole moment of the neutral species, *k*_B_ is the Boltzmann constant, and *T* is the temperature. The *c* term relates to the average orientation of the dipole of the neutral reactant, according to the ratio μ_D_/α^1/2^, and also depends on the temperature of the system.^20^Equation 1 therefore considers several key properties of the polar neutral reactant when predicting the likelihood of the reaction occurring. When comparing the reactions of water and ammonia neutrals with the Kr^+^ ions, these properties are very similar—with the systems featuring comparable dipole moments, polarizabilities, and reduced masses (see Table 2). As such, from a classical capture theory perspective, ammonia and water are expected to react with Kr^+^ at an approximately equivalent rate. It is therefore very interesting that the reactions of Kr^+^ ions with water isotopologues are in excellent agreement with ADO capture theory predictions, whereas the reactions of Kr^+^ ions with ammonia isotopologues simply cannot be accounted for by any capture theory-based models.

A number of other ion–molecule reaction systems (examined under comparable conditions) have reported rate coefficients that are inconsistent with capture theory calculations. For example, a study between ground-state Be^+^ ions and thermal H_2_O, at an effective reaction temperature of 100 K, reported an experimental rate coefficient that was lower than predicted by ADO calculations. A detailed theoretical investigation identified a submerged barrier along the reaction pathway.^34^ The presence of a submerged barrier along the reaction coordinate is known to affect the likelihood of product formation, as previously demonstrated for reactions between laser-cooled Ca^+^ ions and velocity-selected CH_3_F and CH_3_Cl neutrals.^35^ Charge transfer reactions between sympathetically cooled O_2_^+^ or N_2_^+^ and ground- or excited-state Rb atoms, examined in a hybrid ion-neutral trap, reported an interplay between short- and long-range interactions that depends on the quantum state of the reactants. On the basis of *ab initio* calculations, the strength of the non-adiabatic coupling was shown to govern these effects and, subsequently, the agreement between the experimental findings and classical capture theory predictions. The study concluded that, without the aid of high-level calculations, it is not straightforward to predict the behavior of the different charge transfer systems.^36^

What this body of work on ion–molecule reaction systems tells us is that—in the absence of experimental measurements or detailed *ab initio* calculations—it is very difficult to predict whether or not capture theory models can accurately account for the behavior of a given ion–molecule reaction system. Theoretical work on the Kr^+^ + H_2_O reaction system has identified an avoided crossing between the reactant and product potential surfaces, giving rise to efficient (capture-limited) charge transfer. No energetically accessible crossing point was able to be identified between the reactant and product surfaces in the Kr^+^ + NH_3_ reaction system, accounting for the different behavior observed and slower (non-capture-limited) charge transfer.

## Conclusions

Reaction rate coefficients have been measured for the charge transfer reactions between two water isotopologues (H_2_O and D_2_O) and sympathetically cooled Kr^+^ ions. ADO predictions (a classical capture theory model) are in excellent agreement with the experimental findings, indicating that the reactions are governed by a capture mechanism. High-level *ab initio* calculations have identified a crossing between the reactant and product potential energy surfaces, as anticipated from the experimental findings.

Interestingly, charge transfer reactions between ammonia isotopologues and Kr^+^ ions, undertaken under comparable experimental conditions, behave differently to the water reaction systems. The measured ammonia rate coefficients are suppressed in comparison to capture theory predictions, in contrast to the water reactions examined in this work. Additionally, an inverse KIE effect is observed for the ammonia reactions, with ND_3_ reacting faster than NH_3_; the two water isotopologues, instead, show very similar reactivities. High-level *ab initio* calculations confirm that the mechanism driving charge transfer is different when comparing the water and ammonia reaction systems, as no energetically accessible curve crossing was identified for the ammonia reactants. Water and ammonia possess a comparable dipole moment and polarizability, hence they are expected to behave in a similar way (when examined within the framework of capture theory).

Given the lack of experimental data relevant to ISM chemistry, capture theories are often employed in astrochemical models to provide an estimate of rate coefficients of unmeasured ion–molecule reactions. As discussed above, capture theory models do not consistently predict the behavior of a number of ion–molecule reaction systems studied within the framework of Coulomb crystals, even for seemingly “simple” charge transfer reactions. Deviations from capture theory predictions have also been reported when using other experimental techniques, such as CRESU. Hence, the validity of capture theory warrants consideration in each individual reaction system, either via experimental or theoretical means. The need for further work on astrochemically relevant ion–molecule systems is apparent.

## Supporting Information Available

Calculating the partial pressure of water reactants; calculating rate coefficients; secondary reaction pathways; capture theory calculations (PDF)

## Notes

Data Availability: Supporting data can be obtained from DataCat, the University of Liverpool Research Data Catalogue.
