# Supplementary material for: Charge Transfer Reactions between Water Isotopologues and Kr+ ions
Source: ACS Phys Chem Au. 2022 Jan 31;2(3):199–205. doi: 10.1021/acsphyschemau.1c00042 (PMC9136950; doi:10.1021/acsphyschemau.1c00042)
Supplement: Supplementary file 1 — pg1c00042_si_001.pdf [file pg1c00042_si_001.pdf]

Supporting Information: Charge transfer reactions between water isotopologues and Kr^+ ions

Andriana Tsikritea,[1,2] Jake A. Diprose,[2] Jérôme Loreau,[3] and Brianna R. Heazlewood[2,a)]

[1)] *Department of Chemistry, University of Oxford, Physical and Theoretical Chemistry, South Parks Road, Oxford, OX1 3QZ, United Kingdom*

[2)] *Department of Physics, University of Liverpool, Liverpool, L69 7ZE, United Kingdom*

[3)] *KU Leuven, Department of Chemistry, Celestijnenlaan 200F, B-3001 Leuven, Belgium*

(Dated: 23 December 2021)

[a)] Electronic mail: b.r.heazlewood@liverpool.ac.uk

I. CALCULATING THE PARTIAL PRESSURE OF WATER REACTANTS

A complication that needs to be considered when considering the partial pressure of the neutral water reactants relates to the operation of the heat exchange device. The nitrogen heat exchange device is present in the experimental set-up to freeze out contaminant gases and to reduce the pressure of the reaction chamber. After water vapour enters the trap chamber through the high-precision leak valve, it can freeze on the surface of the heat exchange device and be removed from the gas phase. As such, the reliability of the ion gauge readings (the standard method for monitoring partial pressures in Coulomb crystal experiments during the course of a reaction) is not as reliable in this reaction system as in previous studies. The heat exchange device is, nevertheless, necessary to prevent reactions occurring with background gases present in the chamber. To overcome this limitation, the high-precision leak valve is carefully calibrated; water vapour is admitted to the chamber at different leak valve settings, with and without cold N_2 flowing through the heat exchange device. A residual gas analyser is used to establish the partial pressure of water under the different sets of experimental conditions, providing an independent measurement to the pressure gauge reading. Uncertainties in the partial pressure for water are assigned as one standard deviation of all the calibration measurements taken, for each water partial pressure. While this approach results in a higher uncertainty associated with the partial pressure of the neutral reactant than seen in other studies, we wanted to err on the side of caution and to ensure that we fully accounted for the possible sources of error in the measurements.

II. CALCULATING RATE COEFFICIENTS

As detailed in previous work,[1,2] an equation of the form $[H_2O^+]_t = [Kr^+]_0(1 - e^{k't})$ is employed to describe the formation of H_2O^+ ions as a function of time. $[Kr^+]_0$ is the initial number density of Kr^+ ions, and k' is the pseudo-first order reaction rate coefficient. Bimolecular rate coefficients, k, are calculated using $k' = k[H_2O]$, where $[H_2O]$ is the number density of the water reactants. Tens of repeat measurements are conducted for each reactant, at a number of different water partial pressures. The reported uncertainties include both the standard error associated with the fit of the pseudo-first order equation to the experimental data points and the uncertainty in the water partial pressure (see the preceding section).

As noted in an earlier study,[2] the reaction chamber is baked for several days when changing between H_2O and D_2O reactants.

III. SECONDARY REACTION PATHWAYS

H_3O^+ and D_3O^+ ions are seen in the ToF-MS data, alongside peaks arising from the primary charge transfer products (H_2O^+ and D_2O^+). Hydronium is formed when water product ions undergo an additional reaction, abstracting an H (or D) atom from neutral water molecules in the chamber. The hydronium produced by these secondary reactions is also trapped and sympathetically cooled into the crystal. As both water and hydronium have a much lower mass-to-charge ratio than $^{40}Ca^+$, they localise in the dark core of the crystal. Therefore, the total number of product ions present in the dark core does not change if the water ions subsequently abstract an H (or D) atom; while the identity of a product ion may change, the total number of ions in the dark core is dependent only on the charge transfer reaction of interest. (Indeed, given the minimal change to the mass-to-charge ratio of the product ions upon H or D addition, there is no observable difference in the simulated lattice positions of the hydronium or water ions in the dark core of the crystal.)

IV. CAPTURE THEORY CALCULATIONS

Experimental rate coefficients are compared with two further capture theory models, in addition to the ADO calculations detailed in the main text. Briefly, the adiabatic capture centrifugal sudden approximation (ACCSA) model is a rotationally adiabatic quantum capture theory that accounts for the contribution of each individual rotational state to the overall rate coefficient.[3] Details on the calculation can be found in a previous publication.[2] Note that the water molecules are treated as symmetric tops, an approximation that is expected to be valid under our experimental conditions (at temperatures around 240 K).[4] Additionally, a parameterised capture theory model is employed that takes into account the relative kinetic energy of the reaction system at a given rotational temperature of the molecular reactant.[5] The parameterised Su model was developed and tested for a limited range of collision energies—spanning from 300 K to several eV. As such, the collision energies in this work fall outside the range of conditions that the model was developed for. Table I provides

the predicted rate coefficients from these three different capture theory models, alongside the experimental results. While all of the capture theory models involve approximations or extensions of the method (beyond the conditions they were originally designed to account for), the predicted rate coefficients are consistent with the experimental findings: in all of the models and in the experimental measurements, charge transfer occurs with a rate coefficient of approximately $2 \pm 1 \times 10^{-9}$ cm^3 s^{-1}, for both H_2O and D_2O reactants.

TABLE I. Experimental ($k_{\rm exp}$), ADO ($k_{\rm ADO}$), ACCSA ($k_{\rm ACCSA}$) and parameterised Su ($k_{\rm param}$) rate coefficients for the charge transfer reactions between Kr^+ and the two water isotopologues (in cm^3 s^{-1} $\times 10^{-9}$).

Reaction system	$k_{\rm exp}$	$k_{\rm ADO}$	$k_{\rm ACCSA}$	$k_{\rm param}$
$Kr^+ + H_2O$	1.8(7)	1.98	2.98	2.77
$Kr^+ + D_2O$	2(1)	1.90	2.91	2.67

REFERENCES

[1] L. S. Petralia, A. Tsikritea, J. Loreau, T. P. Softley, and B. R. Heazlewood. Strong inverse kinetic isotope effect observed in ammonia charge exchange reactions. *Nature Communications*, **11**, 173, 2020.

[2] A. Tsikritea, K. Park, P. Bertier, J. Loreau, T. P. Softley, and B. R. Heazlewood. Inverse kinetic isotope effects in the charge transfer reactions of ammonia with rare gas ions. *Chemical Science*, **12**, 10005, 2021.

[3] D. C. Clary. Calculations of rate constants for ion-molecule reactions using a combined capture and centrifugal sudden approximation. *Molecular Physics*, **54**, 605, 1985.

[4] T. Stoecklin, D. C. Clary, and A. Palma. Rate constant calculations for ion–symmetric top and ion–asymmetric top reactions. *J. Chem. Soc., Faraday Trans.*, **88**, 901, 1992.

[5] T. Su. Parametrization of kinetic energy dependences of ion–polar molecule collision rate constants by trajectory calculations. *The Journal of Chemical Physics*, **100**, 4703, 1994.
